# Influence of Acidity and Oxidant Concentration on the Nanostructures and Electrochemical Performance of Polyaniline during Fast Microwave-Assisted Chemical Polymerization

**DOI:** 10.3390/polym12020310

**Published:** 2020-02-03

**Authors:** Biwei Qiu, Jingyun Wang, Zhoujing Li, Xia Wang, Xiaoyan Li

**Affiliations:** School of Materials Science and Engineering, University of Shanghai for Science and Technology, Shanghai 200093, China; khvo84@163.com (J.W.); wangxia@usst.edu.cn (X.W.);

**Keywords:** microwave, polyaniline, yield, nanostructure, electrochemical

## Abstract

Polyaniline (PANI), a typical conducting polymer, has attracted great interest as an electrode material. A series of PANIs were prepared through fast microwave-assisted chemical oxidative polymerization with varying HCl and APS concentrations here. It was found that the microwave synthesized PANIs had ~4 times higher for the yields and 7~10 times higher for the electrical conductivity in comparison to PANI samples prepared using conventional method. PANI nanosheets could easily be fabricated in weakly acidic solution due to their oligomeric structure, which contained flat phenazine rings. By contrast, linear PANI chains produced in highly acidic solutions formed nanofibers. The APS concentration did not significantly affect the molecular structures of PANIs under the conditions here. However, increasing the concentration of APS produced nanofibers with shorter branches, which may be due to secondary nucleation during chain growth resulting from increases in active initiation centers. The electrical conductivity and electrochemical performance of PANIs were both improved with increasing HCl and APS concentrations. Improvements due to increases in HCl concentration may be attributed to additions in conjugation length and enrichment of doping levels, while improvements due to increases in APS concentration could be attributed to the increased crystallinity of PANI, which facilitates ion transport.

## 1. Introduction

Energy storage systems, especially flexible supercapacitors [1], have been increasingly explored for their unique properties, such as faster charge−discharge rates, higher power densities, longer life cycles, safer operation, and greater portability, compared with rechargeable batteries and electrical double-layer capacitors. Among the electrode materials for supercapacitors currently available, conducting polymers show the greatest promise for application to flexible integrated electrochemical devices owing to their Faradaic pseudocapacitance effects [2]. The reversible redox reactions of polymers can store additional charges and improve their energy storage performance.

Polyaniline (PANI), a typical conducting polymer, is well known for its multiple molecular structures, reversible doping–dedoping characteristics, good environmental stability, light weight, simple synthesis, and low cost [3,4]. In particular, its large surface-to-volume area, tunable conducting states and doping, render PANI a suitable redox electroactive component and a versatile building block when combined with other capacitive materials, such as carbon nanomaterials [5]. Thus, the reaction conditions for synthesizing various PANIs must be optimized, and the relationships among the structures, morphologies, and electrochemical properties of the resulting materials must be investigated.

PANIs can be prepared through the chemical, electrochemical, or templated polymerization of aniline [6,7]. Optimization of various polymerization conditions, such as the reaction temperature and time, acid and oxidant concentrations, electrolyte, and template, is important to control the polymerization process and tailor the molecular structures and properties of PANIs. Various acids [8,9], such as hydrochloric acid (HCl), sulfuric acid (H_2_SO_4_), benzoic acid (CH_3_COOH), and dodecylbenzene sulfonic acid, and oxidants [10,11,12], including ammonium persulfate (APS), hydrogen peroxide (H_2_O_2_), potassium dichromate (K_2_Cr_2_O_7_), ferric chloride (FeCl_3_) and potassium permanganate (KMnO_4_), are generally used in the chemical and electrochemical syntheses of PANI. Templated synthesis [13,14] provides a route of predictable control to obtain well-ordered geometric structures of PANI endowed by the template itself. This method allows the design of PANI into multiple nanostructures [13,15], such as zero-dimensional nanospheres, one-dimensional nanotubes, nanofibers, and nanorods, and two-dimensional nanosheets. However, PANIs fabricated by electrodeposition or templating are limited by low yields and complex postprocessing. Chemical oxidative polymerization [16], a template-free synthesis, is the major method for producing PANI in high yields. The development of a technique that can provide large-scale quality PANI products with the controllable size and morphology could contribute to break through the restriction by a need for their widespread commercially applications. General temperature control for chemical oxidative polymerization of PANI depends on water bath or ice bath and the reactions usually spend more than 24 h. Recently, microwave-assisted heating has attracted great interest because it is a rapid, energy efficient, uniform, low-cost and environment-friendly heating method. What’s more, microwave-assisted synthesis has been regarded to not only dramatically enhance the reaction rate and yield in PANI syntheses, but also to improve PANI purities by reducing unwanted side reactions [8]. Pandey et al. [17] demonstrated that the microwave-assisted synthesized strategy could even increase the reaction rate by 72 times over the conventional method. Marija et al. [18,19] reported that the yield was almost 3 times higher for PANI synthesized by microwave irradiation comparing to conventional reactor.

The electrochemical performance of PANI is suggested to be highly dependent on its molecular structure and shapes at nanoscale [19,20,21,22,23]. Thus, a deep understanding of the formation mechanism and performance of PANIs is extremely important to improve the design of conductive polymer-based electrode materials for supercapacitors. Yoon et al. [21] analyzed the electrochemical properties of the leucoemeraldine-, emeraldine salt-, and pernigraniline-based forms of PANI nanocomposites with multiwalled carbon nanotubes and found that the specific capacitances of these different PANIs are strongly dependent on their different oxidation states. Li et al. [22] directly prepared PANI onto conducting substrates by electrodeposition and found that the polymer could be converted from nanosheets to nanobelts and then to nanospheres with increasing current density. Electrodes composed of PANI nanobelts showed a larger surface area and higher specific capacitance and stability compared with electrodes composed of nanospheres. Park et al. [23] found that nanofiber electrodes had faster electrode kinetics and better capacitance than nanorods and nanospheres because the former featured outstanding oxidation/protonation levels with structural ordering. Il et al. [24] presented a method for the ice surface-assisted vertical growth of PANI to develop highly conducting two-dimensional PANI nanosheets. Compared with other PANI nanostructures, ice-templated PANI nanosheets showed significantly improved electrical properties, which could be attributed to the long-range ordered edge-on π-stacking of the quinonoid ring. Marija et al. [19] revealed that conductivity values for PANI samples synthesized under microwave irradiation and doped by H_2_SO_4_ were 1.5 times higher than those PANI samples prepared by the conventional method under the same condition.

While considerable progress has been achieved in synthesizing various PANIs, the well-controlled large-scale fabrication of this material with high yield and conductivity remains a difficult research subject. Thus, a simple and efficient route to prepare PANIs with well-controlled morphology and enhanced electrochemical performance must be developed. Microwave synthesis has the potential to address the needs. So, in the present work, PANIs were prepared through microwave-assisted chemical oxidative polymerization using HCl as the acid and APS as the oxidant. The effect of microwave irradiation on the yields and electrical conductivity of PANIs were conducted. Moreover, a facile and quick approach for controlling various PANI nanostructures and synthesis mechanisms was explored by altering the acid and oxidant concentrations of the reaction system. The electrochemical properties of the resulting PANIs were tested to determine their potential application as energy storage devices. Finally, the factors determining the electrochemical performance of the products obtained were explored.

## 2. Materials and Methods

### 2.1. Chemicals and Reagents

Aniline monomers (≥99.5%), *N*-methyl-2-pyrrolidone (NMP), and poly(vinylidene fluoride) (PVDF) were purchased from Aladdin Bio-Chem Technology Co., Ltd. (Shanghai, China). Ammonium persulfate (APS, ≥98%), hydrochloric acid (HCl) (AR grade), organic solvent *N*,*N*−dimethylformamide (DMF, ≥99.5%), sodium sulfate (Na_2_SO_4_) and acetylene black were purchased from Sinopharm Chemical Reagent Co., Ltd. (Shanghai, China). All solutions were prepared with deionized water.

### 2.2. Synthesis of PANI

**Scheme 1:**
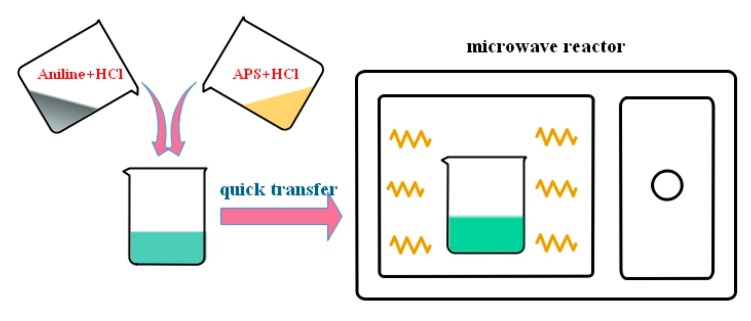
Schematic for preparation of PANI with the microwave-assisted polymerization.

The effects of two factors, i.e., HCl concentration and APS concentration, were investigated during the chemical oxidative polymerization of PANI. First, 0.1, 0.3, and 1 mol/L HCl solutions were prepared. Then, specific amounts of aniline and APS (the ratios of *n*_Aniline_/*n*_APS_ may be referred to in Table 1) were dissolved in 50 mL of HCl in a separate beaker. The two solutions were heated to 50 °C for approximately 10 min in a water bath until a stable temperature was achieved to accurately control the initial reaction temperature. Subsequently, the solutions were rapidly mixed with each other and transferred to a microwave reactor with a preset temperature of 50 °C (temperature mode). The polymerization reaction was triggered quickly by microwave irradiation and sustained for 1 h. Schematic for preparation of PANI with the microwave-assisted polymerization is shown in Scheme 1. The operating procedure could refer to our previous work [25]. To remove impurities and residual acid, PANI products were filtered repeatedly by a centrifugal machine using deionized water, until the pH was neutral. The final PANIs were deposited on the cellulose membranes through vacuum filtration and dried in a freeze drier (Lab-1A-50E, Beijing Boyikang Laboratory Instruments Co., Ltd. Beijing, China) for 12 h. For comparison with PANIs prepared by microwave synthesis, a series of PANIs synthesized with the same proportion of reactants through a conventional method of water-bath at a controlled temperature of 50 °C. Here, the names of sample PANI-0.1H, PANI-0.3H and PANI-1H refer to the PANI products prepared in HCl concentration of 0.1, 0.3 and 1 mol/L respectively and the mole ratio of *n*_Aniline_/*n*_APS_ keeps at 1:0.17 (with aniline concentration of 0.5 mol/L). PANI-1:0.17, PANI-1:0.25 and PANI-1:1 are named for PANI synthesized in 1 mol/L HCl concentration and with corresponding mole ratio of *n*_Aniline_/*n*_APS_ in the Table 1 (with aniline concentration of 0.2 mol/L). They were all obtained by microwave synthesis, as shown in Table 1. As to the conventional method through a water-bath, the names of obtained PANIs added a C as the subscript of PANI, presented in Table 2.

### 2.3. Characterization

The molecular structures of the PANI materials were characterized by Fourier transform infrared spectroscopy (FTIR, Spectrum 100, PerkinElmer Co., Ltd., Waltham, MA, USA) in the range of 450–4000 cm^−1^ and UV–vis spectrometry (UV, Lambda 750, Perkin Elmer Co., Ltd., Waltham, MA, USA) over the wavelength range of 250–800 nm. The dried PANI powders were uniform grinded with potassium bromide (KBr) and then were pressed in pellets before tested by FTIR. For collecting UV–vis spectra, the PANI samples were dissolved in DMF solvent by ultrasonication in advance. The crystalline of dried PANI powders were analyzed via X-ray diffraction (XRD, D8 ADVANCE, Bruker, Madison, WI, USA) using Cu Kα radiation (γ = 0.15406 nm) in the range 2θ = 5°–60°, with a scan rate of 5° min^−1^. To observe the morphology of PANIs, they were deposited on silicon chips and then were dried at 50 °C for 12 h in the oven. After sputter-coated with gold powder, their nanostructures were recorded by using a field emission scanning electron microscope (SEM, Quanta FEG450 FEI Co., Ltd., Hillsboro, OR, USA).

The electrochemical performance of PANI electrodes was conducted on a CHI660E workstation (Shanghai Chen Hua Instrument Co., Ltd., Shanghai, China) in 1 M Na_2_SO_4_, in which PANI electrode, Hg/HgCl_2_ electrode and platinum (Pt) plate were used as working electrode, reference electrode, and counter electrode, respectively. The dried PANI powders, acetylene black conducting agent and a PVDF binder were dissolved in NMP and stirred at the mass ratio of 8:1:1. The mixture was then coated onto nickel foam and pressed by a tablet machine (YLJ-60T, Manufacturing Technology, Inc., Shanghai, China) at 10 MPa, for preparing a PANI electrode. In addition, the pellets of 0.65 mm in thickness and 13 mm in diameter were pressed by the tablet machine. The electrical conductivity (*σ*) of the pellets was measured at room temperature by a RTS-9 4-Point probes resistivity measurement system (Guangzhou 4 Probes Tech, Guangzhou, China).

## 3. Results and Discussion

### 3.1. The Yields and Conductivity of PANIs

To explore the effect of microwave irradiation on chemical polymerization, the yields and electrical conductivity of PANIs prepared with different methods were conducted (Table 2). The chemical oxidation synthesis of aniline with APS was both carried out in aqueous HCl solution with microwave assistance and conventional water-bath. The initial HCl concentration and aniline/APS ratio of the reaction was regulated, as shown in Table 1. Based on the same aniline concentration, the APS concentration increased with increasing the molar ratio of APS to aniline. In Table 2, great enhancement both in yields and electrical conductivity of PANIs for microwave synthesis were found by contrast with that of the products for conventional synthesis. At the same synthesized conditions under microwave irradiation, the values are ~4 times higher for the yields and 7~10 times higher for the electrical conductivity. The result is in accordance with the works published by Marija R. et al. [18,19]. The significant differences between PANI samples synthesized by two approaches implied that microwave irradiation can be used for a rapid synthesis of highly conductive PANIs. The microwave heating bases on the high-frequency reciprocating motion of the dipole molecules inside the heated body. And then, the internal friction heat is generated and the reaction temperature is increased, without any heat conduction, which is necessary for conventional heating. The material inside and outside can be homogeneously heated at the same time. Therefore, rapid intrinsic heating of local spots is responsible for the fast synthesis, which gains more aniline oxidation and high yield comparing to the polymerization at the same conditions through conventional heating. In the meantime, longer molecular chains and conjugated length may generate, resulting in greater electrical conductivity.

The acidity and oxidant concentration both have impact on the yields and electrical conductivity too. As presented in Table 2, there is a trend of major increases in electrical conductivity of PANIs with increasing concentration of HCl and APS, for whether conventional synthesis or microwave-assisted chemical polymerization. As is known to all, electrical conductivity of PANI depends on protonation degree or effective conjugation length of PANI chains [26]. The observed differences here in the conductivity is most probably caused by their molecular structures, which were influenced by the synthesized conditions.

### 3.2. Characterization of the Molecular Structures of the PANIs

In order to investigate the effect of acidity and oxidant concentration on the molecular structures of PANI products during microwave synthesis, characterization and analysis with FTIR and UV−Vis spectroscopy were conducted in this section.

**Figure 1:**
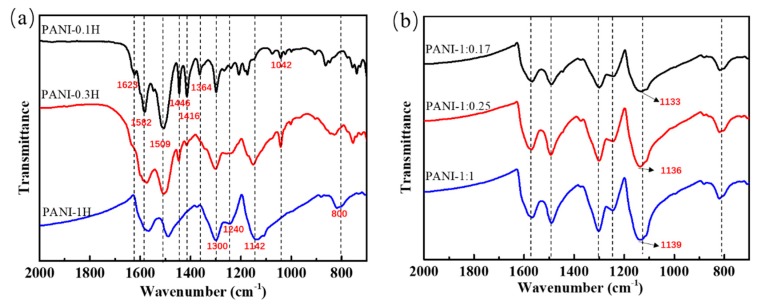
FTIR spectra of PANIs synthesized at different reaction conditions. (**a**) Different HCl concentrations; and (**b**) Different APS concentrations.

The FTIR spectra of the PANIs synthesized at different reaction conditions are shown in Figure 1. Absorption bands located at approximately 1582, 1509, 1300, 1240, and 1142 cm^−1^ correspond to the C=C stretching vibrations of quinonoid (–N=Q=N–, where Q represents the quinonoid ring) and benzenoid (–B–NH–, where B represents the benzenoid ring) structures, C–N stretching vibrations of secondary aromatic amines and BBB units, C=N stretching, and the N=Q=N vibration mode, respectively [8,27]. These peaks represent the main characteristic bands of PANI and can be observed in the FTIR spectra of all PANIs synthesized under the three HCl concentrations adopted in this work. Other absorption bands, such as those at 1623, 1446, 1416, and 1364 cm^−1^, were assigned to the N–H scissoring of primary aromatic amines/phenazine, C=C stretching of aromatic rings, stretching vibrations of phenazine rings, and C–N stretching of phenazine rings; these peaks indicate the formation of aniline oligomers containing phenazine-like segments, which are usually formed by the *ortho* coupling of aniline monomers in the early reaction stage. Characteristic bands representing aniline oligomers were observed in the spectrum of PANI synthesized at 0.1 mol/L HCl solution. These bands weakened when the HCl concentration was increased and even disappeared in highly acidic solution (1 mol/L HCl).

The bands of quinonoid and benzenoid structures shifted from 1582 cm^−1^ to 1567 cm^−1^ and from 1509 cm^−1^ to 1489 cm^−1^, respectively (Figure 1), with increasing acidity of the reaction system. These prominent redshifts may be due to enhanced conjugation interactions and doping of the PANI chains with HCl. The intensity ratio of the quinonoid/benzenoid peaks (*I*_Q_/*I*_B_) could be used to determine the proportion of chain structures to some extent. The calculation results (the values of *I*_Q_/*I*_B_ for PANI-0.1H, PANI-0.3H and PANI-1H are 0.814, 0.875, and 0.914 respectively) revealed that the ratio of quinonoid to benzenoid structures increases with increasing HCl concentration, possibly due to enhanced conjugation interactions.

HCl could be doped into PANI chains in highly acidic solution [8]. For example, the band at approximately 1142 cm^−1^, which is usually found in doped PANI with the N=Q=N vibrational mode, and 800 cm^−1^, which represents the C–Cl stretching vibrations of HCl, could be observed in PANIs synthesized in highly acidic solutions [28,29]. This result indicates that HCl had successfully entered the PANI backbone as a dopant. Doping can cause high electrical conductivity and a high degree of electron delocalization. Moreover, doping provides a partial positive charge that moves the aromatic ring, thereby causing a decrease in the density of aromatic ring electrons. Thus, the vibrational frequency is reduced, and redshifting of the quinonoid/benzenoid peaks occurs. Acid doping may also sometimes occur in weakly acidic reaction systems. For example, the band at 1042 cm^−1^ is a characteristic of the S=O stretching vibration mode of the sulfonated aromatic ring, which is attributed to self-doping by H_2_SO_4_ produced by the decomposition of APS [30].

PANI products synthesized at different APS concentrations showed similar FTIR spectra (Figure 1b), and the characteristic peaks of the quinone and benzene rings remained nearly unchanged. Indeed, only the position of the characteristic peak of the N=Q=N mode was displaced. As the amount of APS increased, the N=Q=N band blueshifted from 1133 cm^−1^ to 1139 cm^−1^, which suggests that conjugation interactions resulting from the quinonoid structure are slightly weakened with increasing APS oxidant. In general, the oxidant can enhance the polymerization rate of aniline and sometimes increase the oxidation state of the PANI products. However, overoxidation affects even the main chains and could lead to chain scission and a concomitant decrease in conductivity [31]. During induction, nucleation depends greatly on the oxidizability of the reaction system. Hence, increases in oxidant concentration may play an important role in chain initiation and accelerate the growth of PANI into short chains under a constant amount of aniline monomers and reaction time. The conjugation length is generally proportional to the PANI chain length, which may explain the observed blueshift in the FTIR spectra.

**Figure 2:**
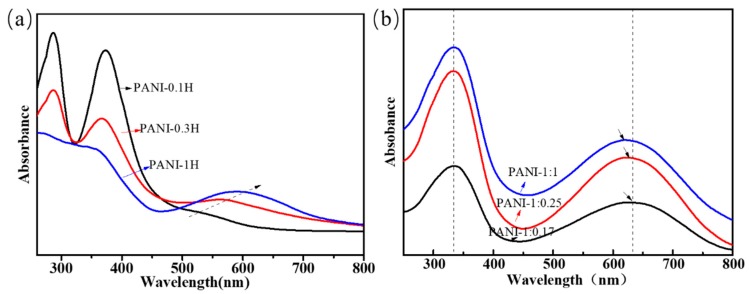
UV−vis spectra of PANIs synthesized at different reaction conditions. (**a**) Different HCl concentrations; (**b**) Different molar ratio of aniline/APS.

Figure 2 shows the UV–Vis absorption spectra of the PANI products. In general, two strong absorption bands at 320–340 and 600–640 nm, which could also be observed in the UV–Vis spectra of PANIs with the emeraldine-based form, were respectively assigned to the *π*–π* excitation of *para*-substituted benzenoid segments and *p*–π* excitation of benzenoid rings into quinonoid rings [32]. In other types of PANIs, such as PANI with different doping states, oxidation degrees, or aniline oligomers, the actual locations of the excitation peaks are believed to depend on the length of the PANI chain and the number of benzenoid and quinonoid rings. The UV–Vis spectrum of the product synthesized in 0.1 mol/L HCl (Figure 2a) shows two strong *π*–π* peaks at 287 and 373 nm and a very weak *p*–π* shoulder at 526 nm (Table 3). The spectrum of the sample synthesized in 0.3 mol/L HCl exhibits a similar phenomenon and reveals two strong *π*–π* peaks at 287 and 367 nm and a *p*–π* shoulder at 563 nm. However, the UV–Vis curve of PANI synthesized in 1.0 mol/L HCl shows only two strong peaks at approximately 340 and 600 nm. The *π*–π* peak located at approximately 290 nm is only observed in some aniline oligomers, whereas the peaks at 350–370 nm may be ascribed to the presence of phenazine or leucoemeraldine-type oligomer structures [8,27]. The blueshifting of the peak indicating *π*–π* excitation (B2) and redshifting of the peak indicating *p*–π* excitation (B→Q) reveal that the phenazine and aniline oligomers are reduced and that the conjugation length of the PANI chains increases with increasing acidity.

The results thus far reveal that numerous aniline oligomers with phenazine-like segments are obtained in weakly acidic solution, whereas long chains of PANI are mainly produced in highly acidic solution. When PANI is synthesized with high concentrations of APS in 1 mol/L HCl solution, two strong absorption peaks located at 333 and approximately 620 nm are observed. While very little difference can be observed in the positions of these peaks, their areas under the curve show remarkable changes. The relative area of *p*–π* and *π*–π* peaks could be calculated to reveal the area of Q/B peaks (A_Q/B_). The A_Q/B_ values of PANIs with *n*_Aniline_/*n*_APS_ of 1:0.17, 1:0.25, and 1:1 were 1.63, 1.42, and 1.27, respectively, and these values decreased with increasing oxidant concentration; this finding shows that the quinone structure in the PANI chains is reduced. The UV spectra obtained are completely consistent with the FTIR analysis.

Taken together, the results reveal that acidity is a more critical factor than oxidant concentration in determining the molecular structures of PANIs. When chemical oxidation polymerization of anilines occurs in weakly acidic solutions, the aniline molecules form aniline oligomers with phenazine-like units via an *ortho* coupling route. Partial ring sulfonation occurs by doping with H_2_SO_4_. However, when oxidation occurs in highly acidic solution, the aniline molecules could be oxidized into anilinium cations and then polymerized to form linear PANI chains via a *para* coupling route. The linkage mode could be confirmed by the band at approximately 800 cm^−1^, which only appears in the spectrum of PANI products polymerized in highly acidic HCl solution. This band originates from the out-of-plane C–H bending of the 1,2-disubstituted ring structure (*para* coupling), which promotes the head-to-tail coupling of aniline during polymerization. The oxidant does not determine the chain growth mode but affects chain initiation during induction.

### 3.3. Morphology and Crystallinity of PANIs

**Figure 3:**
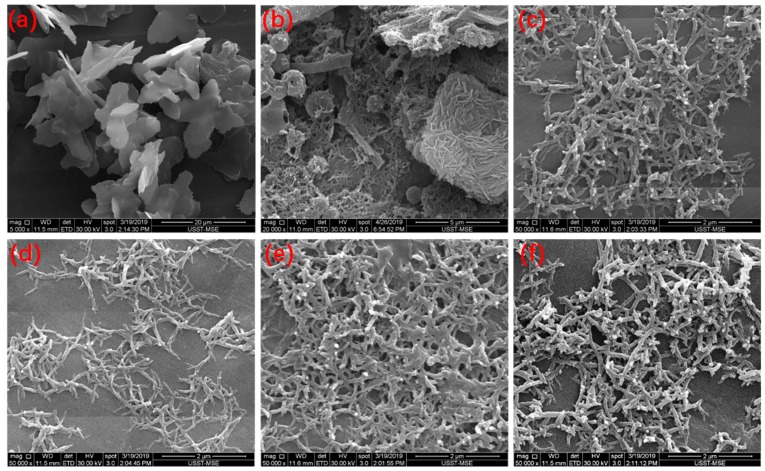
SEM micrographs of PANIs. (**a**) PANI-0.1H; (**b**) PANI-0.3H; (**c**) PANI-1H; (**d**) PANI-1:0.17; (**e**) PANI-1:0.3; (**f**) PANI-1:1.

The nanostructures of the PANIs were observed by SEM, as shown in Figure 3. Sheets with a nanoscale thickness were observed in PANI synthesized in 0.1 mol/L HCl solution (Figure 3a), and nanofibers were obtained when PANI was synthesized in 1 mol/L HCl solution (Figure 3c–f). A mixed and complex morphology of nanosheets, spheres, and short nanorods was observed in products synthesized in 0.3 mol/L HCl (Figure 3b). The morphology of PANI highly depends on its molecular structure. Previous FTIR and UV–Vis analyses revealed that aniline oligomers with heterocyclic phenazine structures are generated in 0.1 mol/L HCl solution. Heterocyclic phenazine rings at the ends of the PANI chains have flat molecular structures, and molecules of this type can produce strong interactions between chains. As a result, these molecules tend to stack and form sheets. Previous FTIR and UV–Vis analyses also showed that PANI molecules, including oligomers with and without phenazine rings and linear chains, are produced when the concentration of HCl is 0.3 mol/L (Figure 3b). As the reaction proceeds, polymerization releases H^+^ to increase the acidity of the reaction solution. Thus, subsequent polymerization with *para* coupling occurs to form linear chains. Short nanorods can result from these short linear chains [3,8]. Further increases in HCl concentration (1.0 mol/L) produce nanofibrous PANIs. This result could be attributed to the accumulation of long linear PANI chains polymerized through *para* coupling to form a fibrous structure. Compared with the short rods in Figure 3b, the lengths and diameters of the PANI nanofibers are greatly increased.

Increasing the APS concentration may result in nanofibers with short branches (Figure 3f). The polymerization reaction begins with oxidation, and the oxidant is highly related to the nucleation capacity of the system. As the APS concentration increases, the increased availability of nucleation sites can cause the polymerization reaction to quickly terminate, leading to the secondary growth of anilines on the surface of the nanofibers.

**Figure 4:**
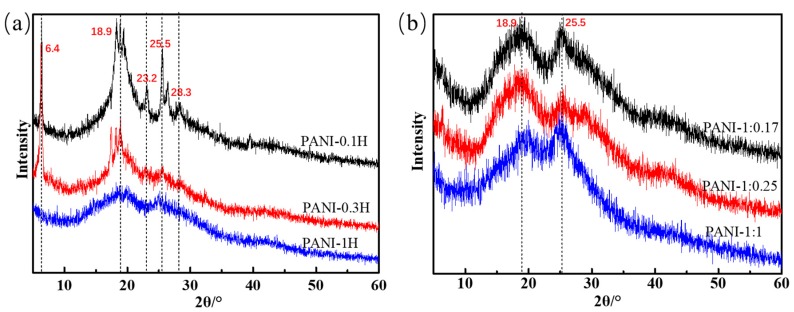
XRD patterns of PANIs synthesized at different reaction conditions. (**a**) Different HCl concentrations; and (**b**) Different molar ratio of aniline/APS.

This morphology can be verified by the XRD patterns shown in Figure 4. The main characteristic peaks of PANI synthesized in 0.1 mol/L HCl are 2*θ* = 6.4°, 18.9°, 23.2°, 25.5°, and 28.3°. The peak at 2*θ* = 6.4° is assigned to the periodicity caused by the dopant acid salt and the N atom on adjacent chains; this peak can only be observed in highly ordered samples [33,34,35]. The peaks centered at 2*θ* = 18.9° and 25.5° are respectively ascribed to the periodicity parallel and perpendicular to the polymer chain. The peaks at 2*θ* = 23.2° and 28.3° may be due to the periodicity caused by *π*–*π* stacking of rigid phenazine-like structures [33]. The presence of aniline oligomers with phenazine-like structures and self-doping by H_2_SO_4_ in weakly acidic systems were previously confirmed by FTIR and UV–Vis spectroscopy. PANI sheets stacked with aniline oligomers with heterocyclic phenazine structures arise from the formation of lamellae between short chains. As the acidity of the reaction system increases, the peaks at 2*θ* = 6.4°, 23.2°, and 28.3° gradually disappear, and the intensities of the main crystalline peaks at 2*θ* = 18.9° and 25.5° significantly decrease; these findings indicate a reduction of oligomers with phenazine rings and less ordering of the PANI chains. The arrangement of nanofiber chains is more disordered than that of nanosheet chains. The XRD patterns of PANIs synthesized at different APS concentrations show two strong peaks at approximately 19° and 25° and a weak peak at 28.5°. This finding indicates that the PANI nanofibers include a few crystals. Increases in APS concentration cause increases in the sharpness of the two main peaks, thus revealing increased crystallinity. This phenomenon may be attributed to the easy movement and rearrangement of short PANI chains.

### 3.4. Synthesis and Formation Mechanism

Studying the detailed molecular structure and morphology of PANI provides a useful means to elucidate the synthesis and formation mechanism of various PANI nanostructures.

In general, the polymerization of PANI features three periods [3]: induction, chain growth, and chain termination. The kinetics of chemical polymerization reveals that induction is sensitive to the initial acidity of the reaction system [8,36,37]. During the early stages of polymerization, APS is rapidly consumed, and exothermic oxidation of aniline occurs. The final molecular structure and morphology of PANI could be strongly related to the linkage type of aniline molecules during induction [36,38]. Aniline dimers and semidines are the first oxidation products. Reaction with the next aniline molecule produces an aniline trimer with linear or coupled linkages. Oxidation of aniline at the *para* or *ortho* position may lead to the formation of linear aniline oligomers or oligomers with phenazine cycles. These species serve as initiation centers and trigger subsequent polymerization. FTIR and UV–Vis spectroscopy have revealed that the fraction of aniline oligomers with phenazine-like segments is high in products collected in weakly acidic reaction solution; however, in highly acidic systems, this fraction gradually decreases and is replaced by polymer chains. When the oxidant concentration is increased and the reaction is maintained in highly acidic solution, the PANI products do not contain oligomers. Hence, oligomerization occurs favorably in weakly acidic solution, and the key factor controlling the *para*-linking of aniline monomers is high acidity but not oxidant concentration.

**Scheme 2:**
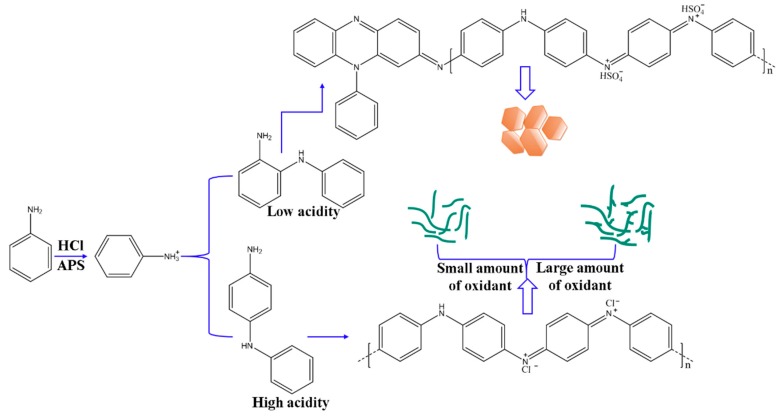
Schematic illustration of aniline polymerization and fabricating PANI nanostructures.

The morphology of PANI is also determined by the synthesis mechanism and its molecular structure. Cyclic phenazine oligomers obtained in low-acidity systems tend to stack on each other and are stabilized by *π*–*π* interactions and electrostatic attraction through self-doping. Highly ordered lamellae consisting of short oligomeric chains are organized into two-dimensional nanosheets. Linear PANI chains produced in highly acidic solutions self-assemble into regular nanofibers due to their preference for disordered entanglement and then aggregate into the cylindrical geometry of nanofibers. When the oxidant concentration is increased in highly acidic solution, nanofibers with numerous short branches or short fibers are obtained. Thus, from the perspective of nucleation, various PANI nanostructures could be formed through homogeneous nucleation, which results in nanofibers, or heterogeneous nucleation, which produces granular particulates [39]. As such, the results obtained are ascribed to a secondary nucleation process. As the APS concentration increases, additional aniline monomers are first oxidized and then evolve into nucleation sites. Nuclei are formed from aniline trimers, and homogeneous nucleation occurs, which leads to the construction of PANI nanofibers. Some new growing points have probably formed on the nascent nanofibers, which can be identified as the secondary heterogeneous nucleation performance. The mechanism of aniline polymerization and formation of PANI nanostructures affected by acidity and oxidant concentration could be illustrated in Scheme 2.

### 3.5. Electrochemical Performance of PANI Electrodes

**Figure 5:**
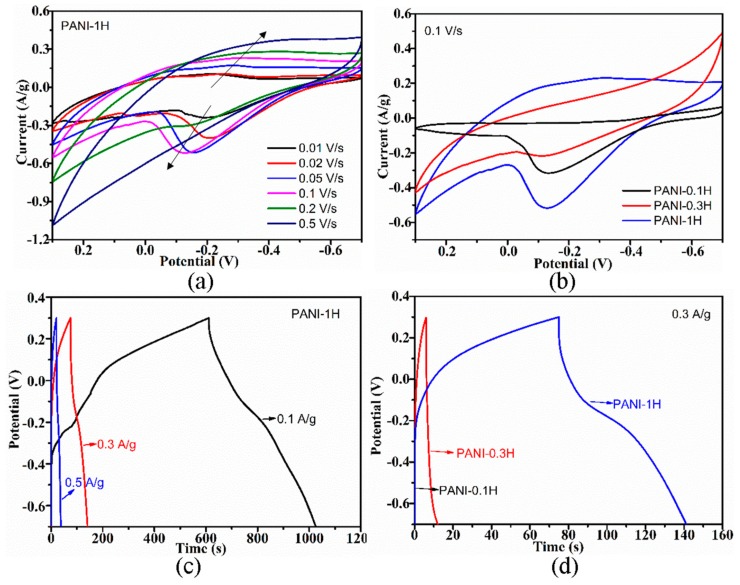
(**a**) CV curves of PANI-1H electrode at different scan rates. (**b**) CV curves of different PANI electrodes at the scan rate of 0.1 V/s; (**c**) Charge-discharge plots of PANI-1H electrode at different current densities. (**d**) Charge-discharge plots of different PANI electrodes at the current density of 0.3 A/g.

**Figure 6:**
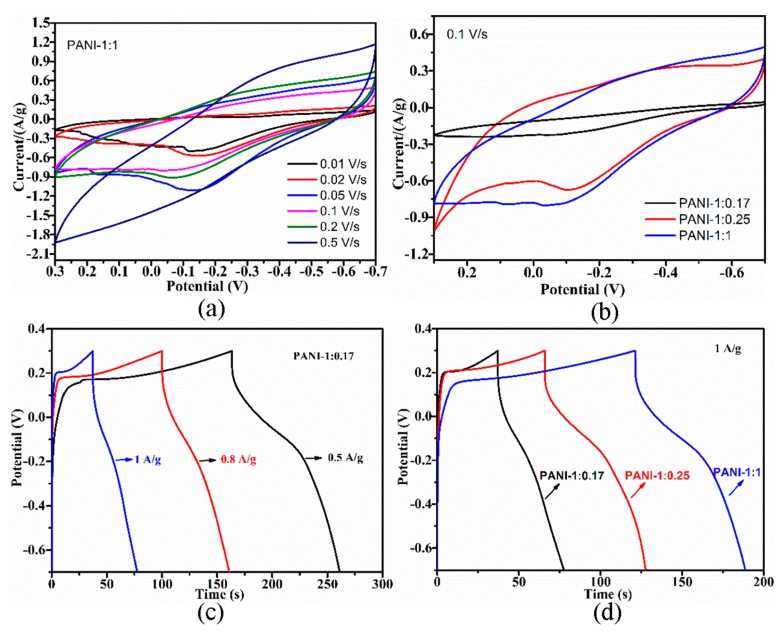
(**a**) CV curves of PANI-1:1 electrode at different scan rates. (**b**) CV curves of different PANI electrodes at the scan rate of 0.1 V/s. (**c**) Charge-discharge plots of PANI-1:0.17 electrode at different current densities. (**d**) Charge-discharge plots of different PANI electrodes at the current density of 1 A/g.

Exploring the electrochemical performance of PANI is necessary to expand its practical application to supercapacitor electrode materials. The cyclic voltammogram (CV) curves of the PANI electrodes were collected at different scan rates, and the corresponding charge–discharge (CD) curves were collected at different current densities. Increases in scan rate resulted in the positive and negative shifting of oxidation and reduction peaks (Figure 5a and Figure 6a), respectively, due to the pseudocapacitance characteristics of PANI and its increasing polarization. At scan rates of up to 0.5 V/s, the redox peaks disappeared, which is ascribed to the greater polarization because the potential of the redox reaction exceeds the scan potential range. The CD curves of the electrode indicated decreases in CD with increasing current density (Figure 5c and Figure 6c), which suggests that PANI has good rate capability, an important characteristic of electrode materials for supercapacitors. Similar CV and CD curves were also obtained from PANI electrodes prepared by using other HCl and APS concentrations.

The CV curves of the PANI electrodes (Figure 5b) showed a larger integrated area with increasing HCl concentration, thus indicating improvements in capacitive performance. In addition, the corresponding CD curves (Figure 5d) measured at the same current density of 0.3 A/g exhibited prolonged CD durations with increasing acidity. The nonlinear shape of the CD curves further implies the pseudocapacitive performance of PANI. Increasing the oxidant concentration improved the capacitive performance of PANI remarkably, which could be confirmed by the observed increases in CV curve area (Figure 6b) and extension of CD time (Figure 6d). Enhancements of electrochemical performance caused by acidity may be due to increases in the conjugation length and enrichment of the doping level of PANI, while improvements of electrochemical performance influenced by APS concentration could be attributed to increases in crystallinity, which facilitates ion transport. The trend of electrochemical performance for PANIs was positively related to their electrical conductivity.

## 4. Conclusions

In this work, a series of PANI nanostructures were synthesized via fast microwave-assisted chemical oxidation polymerization. The reaction conditions, including the acid and oxidant concentrations, were regulated. Microwave irradiation was found to have the potential to prepare PANIs with high yields and electrical conductivity in comparation with conventional method. At the same synthesized conditions for microwave synthesis, the values are ~4 times higher for the yields and 7~10 times higher for the electrical conductivity. The anilines tended to form phenazine-like oligomers, mainly through *ortho*-coupling, in weakly acidic solution. By contrast, PANI with linear chains constructed by *para*-coupling were produced in highly acidic solution. PANI nanosheets were easily fabricated because their oligomeric structures contain flat phenazine rings, while PANI nanofibers were formed by the random piling of linear chains. Thus, nanosheets composed of *π*–*π* stacked rigid phenazine-like structures showed greater order than PANI nanofibers. The electrochemical performance of PANI nanofibers obtained in high HCl concentration was better than that of well-ordered nanosheets because strong acid features enriched doping levels through addition of a protonic acid. APS concentration appeared to have no significant effect on the PANI molecular structure. However, nanofibers with shorter branches were observed with increasing oxidant concentration. The electrochemical performance of the resulting materials improved with increasing APS concentration due to their increased crystallinity, which facilitates ion transport. The results of this work provide a rapid synthesis technology of highly conductive PANIs and better understanding of the relationship between the microstructure and electrochemical properties of PANI for the development of conductive polymer-based electrode materials for supercapacitors.

## Figures and Tables

**Table 1:** The feed compositions of reagents for preparing PANI samples ^1^.

Samples	*C_HCl_* (mol/L)	*C_Aniline_* (mol/L)	*n_Aniline_/n_APS_*
PANI-0.1H	0.1	0.5	1:0.17
PANI-0.3H	0.3	0.5	1:0.17
PANI-1H	1.0	0.5	1:0.17
PANI-1:0.17	1.0	0.2	1:0.17
PANI-1:0.25	1.0	0.2	1:0.25
PANI-1:1	1.0	0.2	1:1

^1^ The ***C*** is abbreviated for concentration and the n represents the mole.

**Table 2:** The yields and electrical conductivity of PANIs synthesized by conventional heating and microwave-assisted chemical polymerization.

Samples ^1^	Yield	*σ* (S/cm)	Samples ^2^	Yield	*σ* (S/cm)
PANI*_C_*-0.1H	10.72%	0.046	PANI-0.1H	43.32%	0.474
PANI*_C_*-0.3H	10.84%	0.078	PANI-0.3H	50.11%	0.755
PANI*_C_*-1H	16.10%	0.338	PANI-1H	58.78%	2.642
PANI*_C_*-1:0.17	15.80%	0.214	PANI-1:0.17	57.43%	1.931
PANI*_C_*-1:0.25	16.10%	0.455	PANI-1:0.25	60.71%	2.425
PANI*_C_*-1:1	25.48%	0.652	PANI-1:1	63.14%	4.575

^1^ The PANI samples were prepared by conventional heating. ^2^ The PANI samples were prepared by microwave-assisted chemical polymerization.

**Table 3:** Characteristic peaks of PANIs investigated using UV–vis spectra.

Samples	Wavelength (nm)		
	B1: *π*–π*	B2: *π*–π*	B→Q: *p*–π*
PANI-0.1H	287	373	526
PANI-0.3H	287	367	563
PANI-1H	-	342	601
PANI-1:0.17	-	335	629
PANI-1:0.25	-	333	623
PANI-1:1	-	333	621
